# Metabolic mapping of glioblastoma stem cells reveals NADH fluxes associated with glioblastoma phenotype and survival

**DOI:** 10.1117/1.JBO.25.3.036502

**Published:** 2020-03-25

**Authors:** Alexandra B. Schroeder, Kelli B. Pointer, Paul A. Clark, Rupsa Datta, John S. Kuo, Kevin W. Eliceiri

**Affiliations:** a University of Wisconsin–Madison, Laboratory for Optical and Computational Instrumentation, Madison, Wisconsin, United States; b University of Wisconsin–Madison, Department of Medical Physics, Madison, Wisconsin, United States; c Morgridge Institute for Research, Madison, Wisconsin, United States; d University of Wisconsin–Madison, Department of Neurosurgery, Madison, Wisconsin, United States; e The University of Chicago, Department of Radiation and Cellular Oncology, Chicago, Illinois, United States; f University of Wisconsin–Madison, Department of Human Oncology, Madison, Wisconsin, United States; g The University of Texas at Austin, Dell Medical School, Department of Neurosurgery and Mulva Clinic for the Neurosciences, Austin, Texas, United States; h University of Wisconsin–Madison, Department of Biomedical Engineering, Madison, Wisconsin, United States

**Keywords:** glioblastoma multiforme, cancer stem cells, fluorescence lifetime, multiphoton microscopy, nicotinamide adenine dinucleotide, metabolism

## Abstract

**Significance:** Glioblastoma multiforme (GBM) is the most frequently diagnosed adult primary brain malignancy with poor patient prognosis. GBM can recur despite aggressive treatment due to therapeutically resistant glioblastoma stem cells (GSCs) that may exhibit metabolic plasticity.

**Aim:** Intrinsic nicotinamide adenine dinucleotide (NADH) fluorescence can be acquired with fluorescence lifetime imaging microscopy (FLIM) to examine its bound and free metabolic states in GSC and GBM tissues.

**Approach:** We compared the mean NADH fluorescence lifetime in live human GSCs and normal neural stem cells and validated those results by measuring oxygen consumption rates (OCRs). We also examined the role that invasive versus less-invasive GSCs had on tumor metabolism by measuring the mean NADH lifetimes and the relative amount of the longer-lived component of NADH and correlated these results with survival in an orthotopic mouse xenograft model.

**Results:** Mean NADH lifetime, amount of bound NADH, and OCR were increased in GSCs. Compared with normal mouse brain, mean NADH lifetimes were longer for all GBM tissues. Invasive xenografts had higher relative amounts of the longer-lived NADH component, and this correlated with decreased survival.

**Conclusions:** FLIM offers cellular resolution quantification of metabolic flux in GBM phenotypes, potentially informing biomedical researchers on improved therapeutic approaches.

## 1 Introduction

Cancer is the second leading cause of death in the United States and one of the most pressing challenges faced by public health institutions.^1^ Approximately 16,830 deaths in 2018 could be attributed to primary malignant brain and central nervous system (CNS) tumors.^2^ It is further estimated that 86,970 new cases of primary malignant and nonmalignant brain and CNS tumors will be diagnosed in the United States by 2019, with 26,170 of those cases coming from primary malignant tumors.^2^ Glioblastoma multiforme (GBM) is a grade IV astrocytoma and is the most aggressive adult primary brain malignancy with a median survival of ∼18  months with maximal safe surgical resection, radiotherapy, and adjuvant chemotherapy with temozolomide.^3^ GBMs are a highly heterogeneous tumor, consisting of bulk tumor differentiated cells, proliferating cells, cancer stem cells, rare clones, resident microglia, and bone marrow-infiltrating immune cells.^4^^–^^6^ While many efforts have been made to understand the metabolic demands of GBM through U251, U118, U87, and other differentiated glioma models,^7^ an increasing number of groups have been investigating the role of stem-like or tumor-initiating cells on the glioma microenvironment to better understand how these cells perturb metabolic adaptations in such a way that make these cells resilient to current therapies.^3^^,^^8^^–^^10^

Poor patient prognosis is the result of recurrence enhanced by the presence of therapy-resistant glioblastoma stem cells (GSCs) remaining postsurgery, facilitating repopulation.^11^ These GSCs create a heterogeneous tumor microenvironment with regions and subtypes of the tumor that become specifically more resistant to current therapeutic methods and may account for tumor recurrence.^3^ Like normal neural stem cells (NSCs), GSCs are marked by their ability to self-renew and yield differentiated progeny. A key characteristic is their ability to initiate tumor xenografts in immunodeficient animal models. Therapeutically resistant GSCs are cancer-initiating cells of tumors after implantation and propagate tumors after serial transplantation.^12^ These cell types contribute uniquely to the understanding of the GBM microenvironment as biological systems rarely display either a purely stem or a purely differentiated composition, and GSCs may also more relevantly maintain the human GBM tumor phenotype.^13^ The presence of these GSCs presents challenges in maintaining tumor control, and importantly, understanding how they may perturb the metabolic environment of the tumor is a crucial step in approaching personalized treatment.^10^

Warburg remarked that most cancer cells rely on glycolysis rather than on oxidative phosphorylation for glucose metabolism—a shift now commonly referred to as the “Warburg effect.”^14^^,^^15^ After glucose is broken down to pyruvate, tumor cells preferentially produce lactate with or without oxygen, even though normal cells undergo oxidative phosphorylation in the presence of oxygen or produce lactate when no oxygen is present.^16^ Cancer cells have been reported to upregulate glycolysis in response to changing oxygen availability and requirements in their environment. However, studies have suggested that the Warburg effect may be an overgeneralization of the metabolic flux and plasticity of cancer cells, particularly cancer stem cells. It was found that human mesenchymal stem cells, which give rise to sarcomas, had an increased dependency on oxidative phosphorylation for metabolism and were less glycolytic than differentiated cells. When these cells were exposed to hypoxic environments *in vitro* or allowed to grow tumors in mice, they switched to glycolysis, but these changes were found to be reversible and did not occur in highly vascularized tumors.^17^ Similar deviations from the well-accepted Warburg cancer metabolism hypothesis have been observed in GBMs. Glioma GSCs exhibit plasticity in response to perturbations in oxygen and nutrient availability and may exploit the pentose phosphate pathway.^10^^,^^12^^,^^18^ Brain tumor-initiating cells derived from U251 cells, while distinct from the human patient GSCs presented here, may also rely mainly on oxidative phosphorylation.^9^ Understanding the role of cancer stem-like cells and their elusive metabolic flux is crucial to treating the tumor in full: if only differentiated cells are targeted with anti-Warburg therapeutics, the cancer stem cells may cause repopulation. Because cancer stem cells impose unique variations in the metabolic profile of tumors, employing imaging techniques and biological assays that are sensitive to these perturbations in the microenvironment may allow for a more quantifiable assessment in a model that merits improved characterization.

Fluorescence lifetime imaging microscopy (FLIM) has been a successful technique for assessing metabolic changes that occur between cancerous and benign cells and tissues.^19^ The metabolic coenzyme nicotinamide adenine dinucleotide (NADH) serves as an electron donor during oxidative phosphorylation, exhibits autofluorescence when excited via single photon excitation at 340±30  nm, and emits light at 460±50  nm.^20^ NADH may be protein-bound or free in the cell, and these states affect the length of fluorescence lifetime, where bound NADH typically exhibits longer lifetimes and free NADH has shorter lifetimes due to the quenching of the adenine moiety.^20^^–^^22^ As cancer metabolism causes shifts in preferred metabolic pathways, the binding of NADH exhibits shifts quantifiable by multiphoton FLIM, which provides a three-dimensional spatial and temporal distribution of fluorophores with high resolution and is sensitive to changes associated with the microenvironment that includes pH, viscosity, temperature, and oxygen concentration.^19^^,^^23^^,^^24^ Skala et al.^19^ observed the shift toward upregulated glycolysis in hamster cheek pouch epithelial cells by reporting a shortening of NADH fluorescence lifetimes and increased amount of free NADH as cells became more neoplastic. FLIM serves as a robust method for observing similar changes in GBMs. Several groups have used fluorescence spectroscopy imaging techniques to observe a shift in mean NADH fluorescence lifetime in GBM tumors versus normal brain in humans.^9^^,^^25^^,^^26^ Trinh et al.^9^ used FLIM to further quantify an increased amount of protein-bound NADH in mice implanted with human U251 tumor-initiating glioblastoma cells compared with tumor regions containing U251 tumor mass forming cells.

The work we present here echoes a similar trend shifting toward longer NADH lifetimes when comparing two scales of models: in human GSC cells versus human NSCs and in GBM xenografts generated from human GSCs versus normal mouse brain tissues. We contrast the metabolic difference in comparing normal stem cells versus cancer stem cells. Furthermore, we characterize different phenotypes from GSCs isolated from human patients that generate tumors that are either more-invasive or less-invasive and show the correlation of metabolic profiling with survival in mice. We show the increase in the amount of the longer-lifetime protein-bound component of NADH in these GBM models. In live cells, we validate FLIM data using a biochemical assay technique (Seahorse XF, Agilent) to directly measure the basal oxygen consumption rate (OCR) in a human “22 GSC” stem cell line and compare it with human NSCs. We further compare the mean fluorescence lifetimes and amount of protein-bound NADH in less-invasive (12.1 GSC, 22 GSC, and 112 GSC), more-invasive (44 GSC and 99 GSC), and U87-derived mouse xenograft models and correlate this with survival. We propose investigating implanted U87 cells to compare the metabolic changes associated with NADH fluorescence lifetime measurements from this classical and differentiated model with our patient-derived GSCs. We use FLIM as a technique to further characterize this stem cell-like niche for GSCs in the tumor microenvironment both in culture and in an orthotopic mouse xenograft model. The technique is sensitive enough to reveal cell-type sensitivity of how GSCs impose a unique metabolic flux within tumors compared with normal, healthy mouse brain tissue, as well as how the metabolism of these stem-cell-generated tumors compare with tumors generated from a wild-type *p53* World Health Organization grade IV malignant glioma differentiated GBM cell line: U87.^7^^,^^27^ To our knowledge, this is the first study to establish correlations with mean NADH lifetime and the relative amount of the longer-lived NADH fluorescence lifetime component ($a_{2}\%$) with human patient-derived GSC phenotype and survival. We propose further characterizing the metabolic plasticity of GSCs as they impose a significant impact on the GBM tumor microenvironment. Offering an enhanced breadth of knowledge to clinicians about the impact of the GSC model may help to further advance targeted therapeutic techniques.

## 2 Materials and Methods

### 2.1 GSC, U87, and NSC Cell Culture

GBM specimens were collected in the operating room under a protocol approved by the University of Wisconsin (UW)–Madison’s Institutional Review Board (IRB). The Health Sciences IRB of the UW–Madison reviewed and approved the protocol prior to collection of any patient specimens under the “exempt” protocol status. The IRB waived the consent requirement because the samples were collected and archived anonymously as “GBM” without identifying information or linkage to patient records. The UW-Madison IRB waived the need for consent as this work was considered “Not Human Subjects Research” by NIH guidelines at the time of collection. GSCs were isolated similarly to previously reported protocols.^28^^,^^29^ Briefly, tumor tissue was collected from surgically resected glioblastoma in the operating room, weighed, minced using a scalpel and chopped twice at 200 μm using a tissue chopper (Sorvall TC-2 Smith-Farquahar). Chopped tissue was plated in suspension at 10mg/ml in growth medium [passaging medium, 20 (PM20): 30% Hams F12, 70% DMEM, 1% penicillin–streptomycin–amphotericin, 2% B27, 20  ng/ml epidermal growth factor, 20  ng/mL basic fibroblast growth factor, 5  μg/ml heparin]. Tumor spheres were apparent within 1 to 2 weeks and then passaged using tissue chopping every 10 to 14 days, as previously described.^28^^,^^30^ Human fetal NSCs were kindly provided by Dr. Clive Svendsen (Cedars-Sinai Medical Center, Los Angeles, California) and maintained as previously described.^31^ Standard serum conditions were used to maintain the U87 cell line (DMEM, 10% fetal bovine serum, 1% antibiotics). For the Seahorse assay and live cell FLIM measurements, 22 GSCs were used and compared with NSCs as a control.

### 2.2 Orthotopic Implantation of GSCs and U87s into Mouse Brains

GBM xenografts were initiated as previously described,^28^^,^^29^ under a protocol approved by the UW Institutional Animal Care and Use Committee. Nonobese diabetic severe combined immunodeficient (NOD-SCID) mice were purchased commercially from Enivgo/Harlan (Madison, WI facility). A total of 18 mice (n=3 for each GBM cell line) were used for GBM implantation and 4 mice that did not receive GBM implantation were used for controls. Animals were housed in dedicated animal vivarium in aseptic caging with a HEPA airflow ventilation system, temperature control, 12-h cycle lighting, and unlimited food and bottle water. Cages included a small animal house and bedding with small paper strips for environmental enrichment, and multiple mice were housed per cage whenever possible. Cages were replaced once per week, and the room was checked daily by trained animal staff with veterinarians on call 24  h/day and 7  days/week. Briefly, GBM GSCs or U87 cells were enzymatically dissociated from single cells, and $2\times 10^{5}\text{ cells}$ or $10^{6}\text{ cells}$, respectively, were suspended in 5  μl of phosphate-buffered saline. Using a Hamilton syringe, cells were stereotactically implanted into the right striatum of anesthetized NOD-SCID mice at 1.0  μl/min at the following coordinates (with respect to bregma point): 0 mm anteroposterior, +2.5  mm mediolateral, and −3.5  mm dorsoventral. At least three mice were examined for each cell line. Anesthesia/analgesic was provided during orthotopic implantation by a cocktail of ketamine (50 to 100  mg/kg) and xylazine (5 to 10  mg/kg), with anesthesia ensured using toe pinch prior to surgery. In addition, a 50:50 mix of lidocaine (2 to 3  mg/kg)/bupivacaine (1 to 3  mg/kg) was administered to the injection site immediately after closing the wound. Postsurgically, buprenorphine (0.05 to 0.1  microgram/g) provided long-term pain control. Animals were monitored daily by research and animal staff until the appearance of neurological symptoms (≈4 to 14 weeks), moribund status, or loss of >15% body weight at which time the animal was immediately euthanized. NOD-SCID mice that did not receive any GBM implantation as controls were euthanized by ${\mathrm{CO}}_{2}$ asphyxiation. Mice were placed in a cage, and 100% carbon dioxide was introduced at the rate of ∼10% to 30% of the cage volume per minute to minimize distress. The death of mice was confirmed by respiratory arrest and the absence of a heartbeat. Brains were then excised, embedded in paraffin, and processed for general histology.

### 2.3 Live Cell FLIM and Seahorse Assay Measurements

The less-invasive stem cell line 22 GSCs and normal NSCs were cultured and passaged as neurospheres in PM20 media. To prepare for the Seahorse experiment, cells were centrifuged and broken up after incubation with 5 ml accutase and counted to ensure consistent confluency. A 96-well plate (Agilent) was coated with 30  μl of laminin per well to maintain stem cell conditions and to improve adherence of the cells to the bottom of the plates.^28^ Two tissue-culture-treated 35 mm #1.5 glass-bottomed imaging dishes (Fluorodish) were also coated in laminin to maintain similar adherent conditions for performing live cell FLIM. In the 96-well plate, 17 wells were plated with 50,000 cells, each of 22 GSCs and 17 wells with NSCs, covered in PM20 media, and incubated overnight at 37°C and 5.0% ${\mathrm{CO}}_{2}$. For the FLIM experiments, 100,000 cells were plated onto laminin-coated glass-bottomed imaging dishes and incubated with PM20 media in identical conditions as the cells in the 96-well plate. One hour prior to the assay, the PM20 media were replaced with unbuffered DMEM (Sigma) with 24 mMol glucose (Fisher) at the suggestion of our collaborators to minimize the antioxidants in the PM20 media affecting the OCR measurements. This step was performed on the imaging dishes 1 hour prior to FLIM to maintain consistent conditions. Live 22 GSCs and NSCs were imaged at n=10 regions of interest for each cell line at 37°C using a Bruker Ultima multiphoton microscope with a Spectra Physics Insight Ultrafast laser. The excitation wavelength was tuned to 740 nm, and an emission filter of 440 (80) nm was used to isolate NADH. Cells were imaged using a 40× water immersion objective (Nikon, NA=1.15) with 4.2-mW incident power. NADH fluorescence lifetime and decay time were captured with H7422P-40 photon multiplier tube (Hamamatsu) and Becker&Hickl SPC-150 time-correlated single photon counting (TCSPC) electronics. The resulting data are fit using the SPCImage software program (Becker&Hickl & GmbH) to a biexponential decay curve, accounting for the different rates of decay for short free ($t_{1}$) and amount of free NADH ($a_{1}\%$), and longer decay time of bound NADH ($t_{2}$) and amount of bound NADH ($a_{2}\%$), as described by Eq. (1). The mean lifetime, $t_{\text{mean}}$, can be calculated by the sum of the products of amount of free with free lifetime and amount of bound with bound lifetime described by Eq. (2).^19^^,^^20^

$$I(t)=a_{1}e^{-t/t_{1}}+a_{2}e^{-t/t_{2}},$$ (1)

$$t_{\text{mean}}=a_{1}t_{1}+a_{2}t_{2},$$ (2)

where $a_{1}+a_{2}=100\%$.

Basal OCRs and extracellular acidification rates were measured using a Seahorse XF24 Flux Analyzer (Agilent) and normalized to background (blank well) readings. Results were averaged and standard deviations were computed using Excel (Microsoft), and a Student’s t-test was performed to establish a significant difference in means between GSCs and NSCs, where p-values <0.05 were considered significant (R Studio). Mean lifetimes and $a_{2}\%$ values for the cells were recorded and averaged using Excel, and a Student’s t-test was used to test for the difference in means.

### 2.4 Fixed Mouse Brain FLIM and Correlation of Mean Fluorescence Lifetimes and Amount of Longer-Lived NADH $a_{2}\%$ with Survival

FLIM was performed on formaldehyde-fixed paraffin embedded samples of mouse orthotopic GSC-derived GBM xenografts. While formaldehyde has been used to reportedly increase fluorescence lifetimes, the metabolic signatures and trends between normal versus cancerous tissues are preserved.^32^ GBM xenografts derived from less-invasive GSCs (12.1 GSC, 112 GSC, and 22 GSC) were compared with highly invasive GSCs (44 GSC and 99 GSC), U87 GBM cell xenografts, and healthy mouse brain (n=3 for each cell line type, n=4 for healthy mice). Sections stained using hematoxylin and eosin (H&E) were used to identify GBM and normal brain areas, and then adjacent (5-μm-thick paraffin cuts) slides of unstained brains were used for FLIM imaging. Tumor xenograft brain sections were imaged using a custom-built multiphoton microscope with a 80-MHz 150-fs pulse-width Spectra Physics DeepSee Ti:sapphire laser as described previously^23^ to excite NADH at 740 nm with 6.2-mW incident power on the sample and capture fluorescence with a H7422P-40 photon multiplier tube (Hamamatsu), a Becker&Hickl SPC-150 TCSPC electronics, a 60× PlanAPO VC oil immersion objective (Nikon, NA=1.40), and a 450 (70)-nm bandpass filter (Chroma). Prior to NADH FLIM imaging, the instrument response function was determined by exciting urea crystals at 740 nm. Photons were counted using TCSPC, and FLIM data were analyzed using SPCImage, fitting NADH decay to the biexponential equation described above and minimizing the chi-squared value to be 1.10±0.05. The instrument response function was measured using urea crystals and deconvolved using SPCImage software. Mean NADH lifetimes and amount of bound ($a_{2}\%$) NADH were fit to the bimodal decay model as described in Eq. (2) and measured for mouse brains that received less-invasive stem lines, mice that received invasive stem lines, mice that received U87 cells, and healthy mice brains (n=15 fields of view for each sample). For all GSC lines and U87 brains, mean NADH lifetime and amount of bound NADH ($a_{2}\%$) within tumor regions were compared with normal mouse brains. A Student’s t-test was performed to determine the differences between cancer and normal tissues with p-values <0.05 considered statistically significant. Mouse survival analysis was performed using a log-rank test and presented as a Kaplan–Meier survival plot with the addition of hazard ratio analysis; p-values <0.05 were considered statistically significant. Plots were generated using GraphPad Prism 6.

## 3 Results

### 3.1 Live Cell FLIM Measures an Increase in Mean NADH Lifetime and Amount of Bound NADH in 22 GSC Line as Validated with a Biological Assay to Directly Measure Oxygen Consumption Rates

**Fig. 1:**
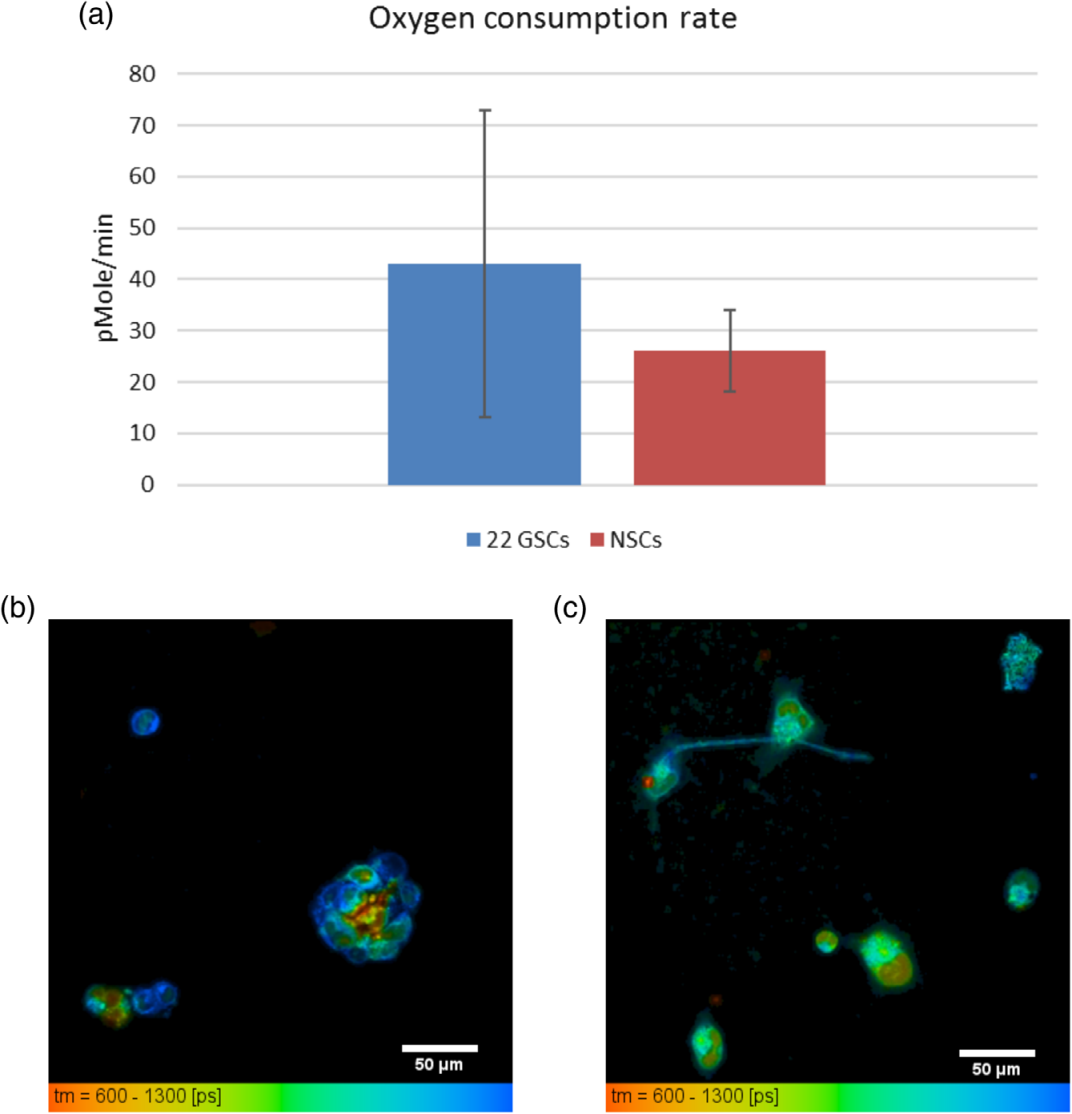
Live 22 GSC cells have increased OCR and corresponding increased mean NADH lifetimes and increased amount of bound NADH ($a_{2}\%$) compared with NSCs. (a) OCR basal measurements taken with Seahorse XF (Agilent) assay by seeding 17 wells with 50,000  cells/well. Mean OCR for 22  GSCs=43.04±29.84  pMole/min versus NSCs=26.10±7.92  pMole/min (Student’s t-test, p=0.0423). Error bars represent standard deviations. FLIM measurements taken on same cell lines with custom multiphoton microscope. Mean NADH lifetimes for (b) 22 GSCs $t_{\text{mean}}=1117.39\pm 106.83\;\mathrm{ps}$, $a_{2}\%=33.0\pm 3.6\%$ versus (c) NSCs $t_{\text{mean}}=1021.73\pm 40.06\;\mathrm{ps}$, $a_{2}\%=30.0\pm 1.5\%$ (Student’s t-test, p=0.005714). Scale bars are 50  μm.

Fluorescence lifetime imaging was performed in tandem with a Seahorse XF24 biological assay (Agilent). This assay technique directly measures the basal OCR of live cells, as well as the extracellular acidification rate (ECAR) of those cells in culture media. The OCR is correlated with oxidative metabolism, whereas ECAR is associated with glycolysis. Results from the Seahorse assay and live cell FLIM are summarized in Fig. 1, which shows that mean OCR was greater in the 22 GSCs (43.04±29.84  pMole/min) than in the NSCs (26.10±7.92  pMole/min, Student’s t-test, p=0.0423) (Fig. 1). Our FLIM measurements of the mean NADH fluorescence lifetime and amount, $a_{2}\%$, of the bound component of NADH reflect agreement with this increase in OCR. The 22 GSC line had a mean lifetime of 1117.39±106.83ps, $a_{2}\%=33.0\pm 3.6\%$ compared with NSCs having a mean lifetime of 1021.73±40.06  ps, $a_{2}\%=30.0\pm 1.5\%$ (n=10 fields of view for each cell line, Student’s t*-*test, p=0.005714) (Fig. 1). This increase in the amount of bound NADH in GSCs, along with the increase in OCR, suggests that GSCs may rely more on oxidative phosphorylation than a normal, NSC control.

### 3.2 FLIM Maps How GSCs Affect the Tumor Microenvironment for Focal and More-Invasive Lines, Compared with U87 and Normal Mouse Brains

**Fig. 2:**
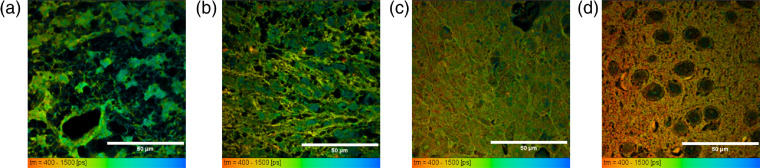
FLIM reveals metabolism impact unique to GSC lines through mean lifetimes in fixed xenograft tissues. Metabolic mapping of NADH is sensitive to showing the difference between more focal tumors (a) 12.1 GSC and (b) more-invasive tumors 44 GSC, (c) U87 nonstem cell tumors, and (d) healthy mouse brain. Mean lifetimes decrease from (a) to (d), reflecting the unique impact that stem cell lines have on the tumor microenvironment. All mean NADH lifetimes of GBMs and amount of bound NADH ($a_{2}\%$) were significantly greater than the normal mouse brain (Student’s t-test, p<0.0001 for both mean lifetime and $a_{2}\%$). FLIM data taken using time-domain collection multiphoton microscope and 60× oil immersion objective (Nikon). Scale bars are 50  μm.

Human patient-derived GSCs were previously described as either producing more focal, less-invasive tumors that remain more localized to one area of the brain or producing more-invasive tumors that spread throughout the brain.^29^ We hypothesized that these stem cell models would yield a different metabolic signature in a mouse xenograft as compared with normal, healthy mouse brain tissue. We further suspected that implanted differentiated U87 cells would have a unique impact on the metabolic microenvironment of GBM xenografts. Figure 2 shows that FLIM creates a metabolic map highlighting the unique impact that less-invasive (12.1 GSC, 112 GSC, and 22 GSC), invasive (44 GSC and 99 GSC), and U87 tumors produce in mice. Mean NADH lifetimes, as well as the amount of bound NADH, varied in all tissues (Table 1). In all GBMs, the amount of bound NADH ($a_{2}\%$) was greater than in the normal brain samples (Student’s t-test, p<0.0001 for both mean lifetimes and $a_{2}\%$), suggesting that GBM tumors may be more metabolically oxidative than normal brain tissue. The invasive lines 44 GSC and 99 GSC present the highest $a_{2}\%$ values compared with the less-invasive tumors. This might suggest that the more-invasive lines have an upregulated ability to flexibly choose to rely more on oxidative phosphorylation pathways, potentially contributing to their resilience and poor prognosis.

**Table 1:** Mean NADH fluorescence lifetimes and relative amount of bound NADH in GBM mouse xenograft tissues compared with normal mouse brain.

Xenograft type	GBM mean fluorescence lifetime (ps)	Normal mean fluorescence lifetime (ps)	GBM percentage bound NADH (%)	Normal percentage bound NADH (%)
12.1 GSC (focal)	1054.56±169.56	663.83±150.06	29.14±3.40	24.39±2.55
112 GSC (focal)	892.50±186.80	663.83±150.06	31.66±4.73	24.39±2.55
22 GSC (focal)	852.24±173.44	663.83±150.06	29.65±2.94	24.39±2.55
44 GSC (invasive)	883.81±136.99	663.83±150.06	32.38±2.01	24.39±2.55
99 GSC (invasive)	880.46±114.43	663.83±150.06	33.80±3.25	24.39±2.55
U87 (nonstem cell)	850.76±175.13	663.83±150.06	30.10±2.50	24.39±2.55

### 3.3 Increase in Mean NADH Lifetime is Associated with Improved Survival in Mice with Focal GSC Xenografts, but Increase in Amount of Bound NADH is Associated with Decreased Survival

**Fig. 3:**
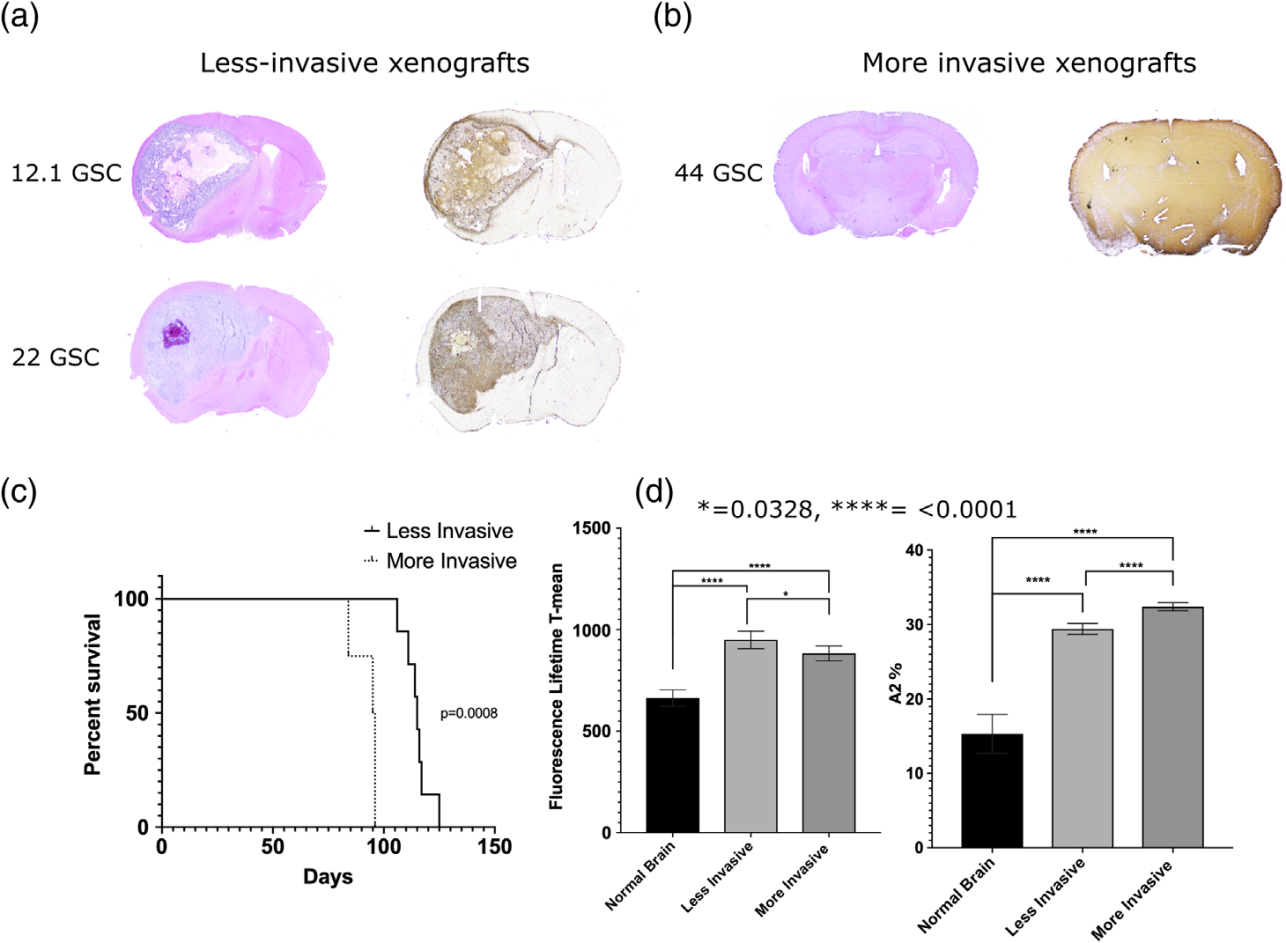
Longer NADH fluorescence lifetimes associated with focal GBMs correlate with improved survival in mice. H&E stains, left, coupled with Vimentin, right differentiate between (a) focal xenografts and (b) invasive xenografts. (c) Kaplan–Meier curve showing that mice with less-invasive xenografts had improved survival (log-rank test, p=0.0008), (d) which correlated significantly (log-rank test, p<0.0001) to longer NADH fluorescence lifetimes. There were significant differences (log-rank test, p<0.0001) between the amount of longer-lived fluorescence lifetime of NADH ($a_{2}\%$) between normal, less-invasive, and invasive xenografts that correlated with survival.

Previously, Pointer et al.^29^ reported a significant association of collagen architecture with GBM survival in mouse xenografts. Mice that had focal, less-invasive tumors overall had more organized collagen with wider fibers, smaller mean angle between fibers, and longer and straighter fibers compared with collagen in invasive xenografts.^29^ Based on evidence of collagen structural changes being prognostic in GBMs and in other cancer models such as breast^33^^,^^34^ and ovarian cancer,^35^ we wanted to further examine if there was a significant survival association with mean NADH fluorescence lifetimes and these two groups of tumor phenotypes. After establishing that the NADH fluorescence lifetimes varied between cancerous and normal tissue, we assessed the survival impact of these different mean lifetimes by grouping two of the less-invasive tumor-generating lines (12.1 GSC and 22 GSC) and comparing them with an invasive tumor-generating line (44 GSC). The mean NADH lifetime of the two less-invasive line tumors was 949.83±198.48  ps, and the mean NADH lifetime of 44 GSC tumors was 883.81±136.99  ps (Student’s t-test, p=0.0214). These values reflect that less-invasive tumors tend toward longer mean NADH lifetimes. Mice with less-invasive tumors (12.1 GSC and 22 GSC, n=3 for each line) had a significantly better survival probability than mice with invasive tumors (44 GSC, n=3) (log-rank test, p=0.0008) and a median survival of 115 days for mice with focal tumors (12.1 GSC line: 116.5 days, 22 GSC line: 111 days). Mice with the invasive line tumors (44 GSC) had a median survival of 95.5 days. The mouse survival analysis was performed using a log-rank test and presented as a Kaplan–Meier plot, where p-values <0.05 are considered statistically significant (Fig. 3).

## 4 Discussion

The observed preferential metabolic shift of cancer cells toward upregulated glycolysis has been a well-accepted hallmark of cancer since Otto Warburg coined the Warburg effect in 1956. While many epithelial cancer cells and tissues, as well as differentiated GBM tissues, exhibit upregulated glycolysis, particularly in the context of hypoxia,^19^^,^^36^^–^^42^ there is evidence to suggest that cancer stem cells may exhibit a more flexible choice in preferred metabolic pathway that is dependent on the surrounding tumor microenvironment.^8^^,^^9^^,^^18^ Our measurements of live cell assays directly compare cancer stem cells versus normal NSCs, showing the difference in oxygen consumption being enhanced by cancerous GSCs compared with normal NSCs. These measurements are reflected in both the live cell and tumor tissue fluorescence lifetime measurements of NADH, revealing an increase in the longer-lived $a_{2}\%$ component or amount of bound NADH and a shift toward longer mean fluorescence lifetimes compared with NSCs and normal mouse brains. The mean NADH lifetimes are themselves unique to the human-derived GSC line, where the less-invasive xenografts produce longer mean lifetimes than the more-invasive xenografts and U87 xenografts. In particular, our measurements of the $a_{2}\%$ longer-lived fit component are highest in the invasive xenografts compared with the U87 xenografts, less-invasive xenografts, and normal brain. Correlations with the amount of NADH in U251 glioma cells and mobility showed that lower available amounts inhibited invasiveness,^43^ so further investigating the metabolic flux of NADH in less-invasive versus invasive stem cell lines and potential consequences for survival is merited. While overall mean NADH fluorescence lifetime increases in all GBM cancer models that we have explored compared with normal mouse brain tissue or NSCs, it is worth noting that the increase in $a_{2}\%$ of the longer-lived NADH component of the more-invasive GSC line might be indicative that these more-invasive stem cells produce a phenotype that has an enhanced ability to metabolically utilize oxidative phosphorylation or be flexible in pathway choice compared with the less-invasive GSCs with overall longer mean NADH fluorescence lifetimes. In this way, it could produce a more resilient lineage that contributes to decreased survival.

To our knowledge, this is the first study that has compared GSCs with a normal NSC control for live cell studies. We metabolically profile different phenotypes of GSCs and report on the changes to NADH fluorescence lifetime and relative amount of bound NADH. Each individual less-invasive, invasive, and U87 cell line has been shown to produce regions of tumor mass that contain an increase in amount of bound NADH, or $a_{2}\%$, compared with normal brain as shown in Table 1 (Student’s t-test, all p-values <0.05). Furthermore, more-invasive stem cell lines (44 GSC and 99 GSC) reveal an increase in the amount of bound NADH compared with less-invasive stem lines (12.1 GSC, 112 GSC, and 22 GSC) and U87 lines as shown in Table 1 (Student’s t-test, $p=3.24\times 10^{-12}$). The amount of bound NADH in less-invasive cell lines combined is significantly different than the amount of bound NADH in the combined invasive stem cell lines (Student’s t-test, $p=8.271\times 10^{-10}$); however, it does not vary significantly when compared with the U87 cell line generated tumors (Student’s t-test, p=0.7691). U87s generate a tumor mass of bulk differentiated cells that reside mainly on one side of the brain; less-invasive stem cell lines also generate tumors that exhibit the similar characteristic of producing a more focal and localized tumor. While the U87 glioma cell line has been a long and widely used cell line, it has been recently described through genetic testing that U87 cell lines have been misidentified and misrepresentative of the tumor of origin.^44^ U87 cells grown in serum deviate from the tumor tissues from which they may have been harvested, and while still likely that U87’s are derived from glioblastoma, the genetic profiling does not match with the original patient specimen.^45^ The amount of bound $a_{2}\%$ in invasive cell lines does vary significantly when compared with the lower amount of bound in U87s (Student’s t-test, $p=6.325\times 10^{-10}$). To our knowledge, this work is the first to establish a correlation with increased mean fluorescence lifetime and $a_{2}\%$ seen in less-invasive GBMs and improved survival in mice.

Current therapeutic strategies primarily target differentiated tumor cell traits, often leaving behind resistant GSCs that account for poor therapeutic response and tumor repopulation. FLIM offers the sensitivity to discern the cellular microenvironment as impacted by GSCs versus differentiated glioma cells, as well as normal NSCs and normal brain tissue. Cellular metabolic information provided by FLIM can be coupled with either *in vivo* photoacoustic measurements of glucose uptake in the mouse brain^46^ or magnetic resonance spectroscopy of C13-pyruvate to track metabolic changes across multiple spatial scales.^47^ In addition, Shah et al.^48^ successfully used NADH FLIM parameters, such as mean lifetime and amount of free NAD(P)H, to quantify cellular heterogeneity across cells and to track the metabolic response to chemotherapy treatment. GBM is a complicated heterogeneous cancer model with a hierarchy of subtypes and different cell populations, and we propose that characterizing the GSC influence on metabolic plasticity provides a more comprehensive understanding of this cancer. Through metabolic profiling of GSCs, we expose their unique adaptations to the cancer stem cell microenvironment and their impact on metabolic flux. Successful therapeutic approaches require targeting properties, not only of differentiated GBM cells but also of the GSCs. By exploiting the nuanced metabolic properties of stem versus differentiated GBMs, clinicians may be able to tailor therapies to account for metabolic plasticity between cells.

## 5 Conclusions

Through the use of NADH FLIM, we have metabolically profiled the changes associated with GSCs in the context of both cells versus normal NSCs and unique phenotypes of GBM tumors in mouse xenograft models compared with normal mouse brains. GSCs exhibit a longer mean lifetime and increased amount from the longer-lived bound NADH as well as express a higher OCR as measured with a Seahorse assay. We have shown that GBMs also exhibit longer mean NADH fluorescence lifetimes than normal brain, with an increase in the contribution from longer-lived bound NADH across both invasive and less-invasive GSCs, as well as a U87 tumor xenograft model. Finally, we have correlated this marked ability of GSC-based GBMs to utilize oxidative metabolic pathways with survival in the same mouse xenograft model, showing that more-invasive GSCs with the highest contribution from bound NADH have a significantly decreased survival.
